# On Analyzing Routing Selection for Aerial Autonomous Vehicles Connected to Mobile Network

**DOI:** 10.3390/s21020399

**Published:** 2021-01-08

**Authors:** Jordi Mongay Batalla, Constandinos X. Mavromoustakis, George Mastorakis, Evangelos K. Markakis, Evangelos Pallis, Tomasz Wichary, Piotr Krawiec, Przemysław Lekston

**Affiliations:** 1 Department of Telecommunications, Warsaw University of Technology, 00-661 Warsaw, Poland; tomasz.wichary.dokt@pw.edu.pl; 2 Department of Computer Science, University of Nicosia, 24005 Nicosia, Cyprus; mavromoustakis.c@unic.ac.cy; 3 Department of Management Science and Technology, Hellenic Mediterranean University, 72100 Crete, Greece; gmastorakis@hmu.gr; 4 Department of Electrical and Computer Engineering, Hellenic Mediterranean University, 72100 Crete, Greece; emarkakis@hmu.gr (E.K.M.); pallis@pasiphae.eu (E.P.); 5 Department of Internet Services and Applications, National Institute of Telecommunications, 04-894 Warsaw, Poland; p.krawiec@il-pib.pl; 6 FlyTech UAV Sp. z o.o., 30-149 Kraków, Poland; plekston@flytechuav.pl

**Keywords:** machine learning, two-phase selection algorithms, UAV, routing and forwarding, optimization

## Abstract

This paper proposes a two-phase algorithm for multi-criteria selection of packet forwarding in unmanned aerial vehicles (UAV), which communicate with the control station through commercial mobile network. The selection of proper data forwarding in the two radio link: From UAV to the antenna and from the antenna to the control station, are independent but subject to constrains. The proposed approach is independent of the intra-domain forwarding, so it may be useful for a number of different scenarios of Unmanned Aerial Vehicles connectivity (e.g., a swarm of drones). In the implementation developed in this paper, the connection is served by three different mobile network operators in order to ensure reliable connectivity. The proposed algorithm makes use of Machine Learning tools that are properly trained for predicting the behavior of the link connectivity during the flight duration. The results presented in the last section validate the algorithm and the training process of the machines.

## 1. Introduction

Unmanned aerial vehicles (UAV) put several challenges to the communication technologies due to strict requirements on quality of the connection and, specially, on reliability [1]. In addition, connectivity is also at risk due to physical scenario with open air and long distances between nodes, whereas one positive feature for assuring good quality of transmission is the low number of physical obstacles in the air. Many efforts have been dedicated to find optimal routing for communication between nodes, where the nodes may be UAVs and base stations and compose the so-called aerial Wireless Sensor Network (aerial WSN). Many of the algorithms for routing inside aerial WSN use the well-known Ad-hoc On-demand Distance Vector (AODV) [2,3] routing, however routing algorithms such as Optimized Link State Routing (OLSR) can be also implemented. The handicap of AODV is that the path discovery requires a high amount of time for the establishment of the initial forwarding routes [4]. On the other hand, link state routing sends to all the network nodes information about the neighbors, which implies that many routing messages must be sent for the establishment of forwarding routes. Moreover, Leonov and Litvinov showed that high dynamicity makes the use of OLSR difficult [5].

The communication technologies of drones consider two main scenarios: (1) The UAV connects directly with the control station and (2) the UAV connects with other UAVs as intermediate hopes to the control station (so-called UAV swarms [6,7]). For direct communication between UAV and control station [8], there are two main technologies: The first one uses 433 MHz unlicensed band and has a limitation of about 2 or 3 km of distance between the control station and the UAV [9]. The other is based on commercial mobile networks that use licensed (reserved) bands for reliable communication. Mobile networks (3G/4G/5G) are used in the case that 433 MHz-based technology cannot be used for different reasons such as interferences, maximum distance, and unavailable technology.

In communication through mobile network infrastructure, the UAV must maintain direct connectivity with the mobile operator (network operator) [10]. With this scope, aerial WSN nodes have, in general, omni-directional antennas; however, ground antennas may be both omni-directional or with beamforming technology. This technology consists of pointing the radioelectric beam in the direction of the UAV, which reduces the energy used in transmission [11]. In order to ensure reliability of communication between UAV and mobile network, parallel routing should be provided, while leaving to the UAV the possibility of selecting the most appropriate routing path and, preferably, connection adaptation should be possible in the case that current connectivity losses quality.

If the UAV has several paths that may serve a connection, one of the tasks of the communication system is to select the most appropriate path for a given connection [12]. This selection should be based on the current parameters of the network, and, specifically, on the current parameters of each potential path [13]. This multi-criteria selection of routing is an optimization problem that is, in general, *NP*-complete.

Several approaches have been taken to solve multi-criteria routing. One of the newest ones is the use of Machine Learning approaches to predict the behavior of the network based on current measurements. This is the approach used in this paper. We introduce a two-phase routing selection implemented with the use of Machine Learning algorithms. The two phases refer to the two necessary routing selection in the following links: (1) The link between the UAV and the mobile network base station and (2) the link between the base station and the control station (controlling the UAV flight). Our study is based on a measurement analysis of the connectivity between UAV and control station through mobile networks. The main contributions of this paper are: (1) A practical solution based on real measurements of UAV using mobile network, (2) the introduction of two-phase Machine Learning (ML) tool that makes use of Utility Theory for coherent connection of both ML phases. Two-phase ML connected through a Utility formula can be extended to many other applications, and (3) the introduction of a combination of different prediction algorithms for better estimation of the resources in mobile networks.

In the Section 2, we present the related work including the principles of multi-criteria selection, whereas in Section 3 we show the motivation of our system for routing selection. We performed several tests in UAV connected through LTE and we argue that one connection (through one network operator) risks to fail in any moment during the connection if this is not monitored. We propose our solution based on two-phase multi-criteria selection of routing path. In Section 4 our system is formally described based on Utility theory. Section 5 presents the measurements taken on the network as well as the machine learning training and validation. At last, the paper is concluded in Section 6.

## 2. Related Work

Routing selection in current networks requires multi-criteria selection algorithms, i.e., a selection based on optimization of a number of parameters. Multi-criteria optimization is achieved by defining a decision space that includes all the potential solutions that could result from the process of decision. Such potential solutions are called decision vectors. Each vector is *m*-dimensional, where *m* is the number of variables. Then, the decision space defines the feasible solutions of each one of the variables. The multi-criteria decision process aims to maximize or minimize a number (call *k*) of objective functions. All the *k* objective functions are called together the aggregate objective function.

With the above definitions, a solution X dominates a solution Y when achieves better results in all the *k* objective functions (maximized or minimized). A solution X is called efficient if and only if there is not any other solution that dominates it. All the efficient solutions together are called Pareto Optimal and their outcome vectors are called the Pareto Frontier. If the Pareto Optimal contains only one solution, than the decision process is obvious, however, normally the Pareto optimal contains several efficient solutions and the multi-criteria decision process needs to provide some policy to take the final decision.

Different policies may provide more information a priori, i.e., before analyzing the Pareto optimal, or a posteriori, i.e., the decision algorithm may introduce some information after the analysis of the Pareto optimal, such that the final Pareto optimal will contain one efficient solution [14]. For reducing the number of efficient solutions to one, a normal approach is to propose a function to reduce the multi-criteria problem to one unique variable. Such a function is named cost function [15]. One of the most common cost function is the Minkowski norm of order *p*. Minkowski norm is non-linear, so the aggregation (the reduction of a number of efficient solutions to one) is also non-linear. A non-linear aggregation is able to come closer to the optimized solution subject to the fact that the algorithm has information about the order *p*, otherwise non-liner aggregation may never reach optimized solution. In addition, it is not possible to know whether the solution specified by the algorithm is better or worse than other solutions, unless the Pareto Frontier is compared for all the Pareto optimal (which often is unworkable).

There are other propositions to the cost function that aim to reduce the number of efficient solutions (the Pareto optimal). The decision algorithms with reference levels limit the number of solutions by defining two levels for each variable, one that should not be exceeded by any selected efficient solution (called reservation level) and one that once exceeded by an efficient solution, then any better value should not impact in the final decision (called aspiration level) [16,17]. In [18], the authors adjust the reference levels to the decision space, such that the distance of the reference levels to the center of mass of the solutions is minimized. An extended approach of the decision algorithm with reference levels consists of using the Mahalanobis distance instead of the Euclidean distance [19].

## 3. Motivation

Our solution is based on multi-criteria routing selection. In this section we argue that such a solution is required when we assume communication of the drone through commercial mobile network. In Section 3.1 we describe the scenario taken into account and provide message delay measurements of drones communicating in commercial mobile network. The results show the necessity of multiple routing paths for assuring reliable communication. In Section 3.2, we present the solution adopted in this paper.

### 3.1. Description of the Scenario and Tests of Communication Based on Mobile Network

Our scenario includes an unmanned aerial vehicle Birdie UAV that develops transmission links established through 4G mobile network. In particular, mobile communication covers transmitting control and telemetry signals based on a compact 4G modem with USB 2.0 interface with dimensions that should not exceed 100 × 30 × 15 mm and maximum weight of 40 g.

The additional control station (also called ground station) is equipped with Mission Manager software and a configured VPN client that allows to establish a secure, encrypted connection to the drone over any internet connection (overlay encryption). The control station is also connected to the Internet through a mobile network. So, our scenario contains two radio links: On the one hand UAV to antenna, and, on the other hand, control station to antenna. Normally, these two radio links are not situated in the same segment of the antenna [20] (one segment is pointing to the air and the other pointing to the ground), so we can assume that the two radio links are independent as far as resource usage is concerned.

MAVlink communication (telemetry and control) is transmitted directly through UDP streams. The level of network latency shown in the current (4G) generation of mobile networks was around 200 ms (average), which is acceptable for controlling the implementation of UAV missions [21]. It is assumed that the flight works in the supervisory mode, i.e., without the necessity of direct control of steering surfaces by the operator. Flight modes such as FBWA or CRUISE can be safely used to maintain the link latency at a level not exceeding 300–500 ms, while for flight modes such as LOITER, RTL, AUTO, and GUIDED even delays of 1–2 s still allow a secure mission execution. It has to be underlined that implementation of this type of operations requires the presence of a VLOS operator to carry out the drone’s launching and landing processes.

The quantitative evaluation of communication link quality has been limited to monitoring of link delays. For the tests, the UAV is equipped with NanoPi Neo Core 2 on-board computer, based on 4 core ARM Cortex A53 processor with clock frequency up to 1.5 GHz. The load of the processor during work with the first version of the software was about 25% (i.e., 100% on one of the 4 cores). After re-designing the on-board software, it was possible to reduce processor consumption to about 4–5% in a continuous operation mode [22]. This optimization was particularly important due to the avionics chamber heat management. The whole range of navigation functions as well as the current monitoring of the state of the airborne components were carried out by the Autopilot module with integrated sensors. The hardware platform is Pixhawk version 1 (PX4 v2 project with ETH Zurich), providing support for all peripherals based on the NuttX OS real-time system. The software used for flight is a modified ArduPilot package with “PX4 Middleware” libraries. The communication with the mobile network is ensured by the installation of MF823 on-board modem, which provides support for 2G/3G/4G networks [23]. The modem is mounted in a chamber under the hatch of the battery, while maintaining the ability to access the modem on the ground without having to cut the power supply from the Birdie UAV set. The status of the network connection is signalized by a LED that indicates the availability of the network. Possible connection statuses according to the status of the LED are: (1) Red: modem on, but not logged into any network, (2) Green blinking: Modem connected to LTE network, (3) Green continuous: Modem logged into LTE network, (4) Blue blinking: Modem connected to 2G/3G network, and (5) Blue continuous: Modem logged into 2G/3G network.

In order to connect to the plane, the following steps had to be done: (1) Run the Birdie system and check the status of the multicolored diode on the modem (recommended green for LTE connection, allowed blue for 3G connection, red for no cellular network coverage); (2) connect the computer to the Internet (e.g., through a pre-set 3G/4G hotspot), and (3) after about 2 min from the powering of Birdie system, activate the connection from the desktop, open the Mission Manager application and select the connection option on port 14,550 from the Connection menu.

**Figure 1:**
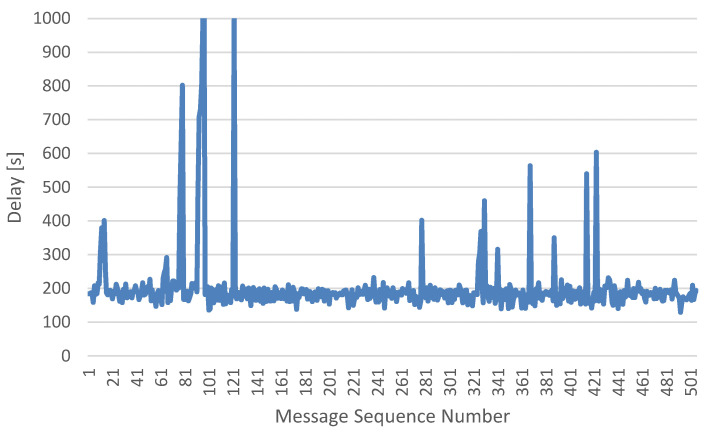
Delay measurements in communication between drone and control station.

The test results showed that in an urban environment, the average of the delay was around 200 ms, with possible delays of up to 500–800 ms. Values of delay higher than 1 s were already classified as temporary loss of communication. In case of temporary loss of communication, the Birdie system was equipped with “Fail-Safe” functionality, where the user defines (during the preparation the plane before flight) the maximum allowed time without communication and activates “GCS Failsafe” (by selecting RTL option). Figure 1 shows the delay results for the testing drone.

These preliminary tests showed that latency is one of the main factors impacting the quality of the connection and poor quality connection risks the manageability of the UAVs. The throughput of the connection is, under normal conditions, low: MAVlink connection requires about 3 Kbps of capacity, however cyclic transmission of a flight plan may require up to 15–20 Kbps. The last important parameter impacting the communication with UAV is the reliability: Disconnecting and re-establishing connection through the mobile network on the fly involves downloading the full set of about 900 autopilot parameters. Therefore, possibility of additional and monitored routing is necessary for providing secure and reliable communication between the control station and the UAV.

### 3.2. Description of the Solution

**Figure 2:**
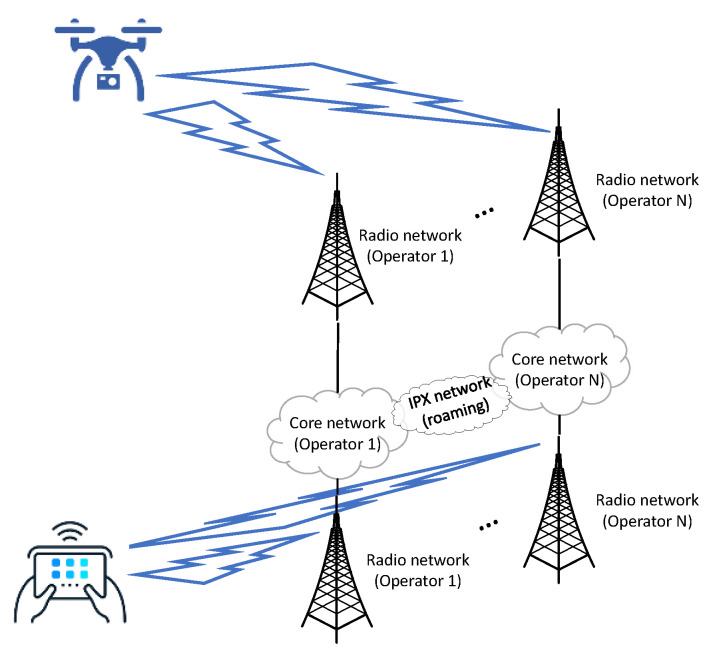
Connectivity scenario of two links: Unmanned aerial vehicles (UAV) to antenna and antenna to Control station. *N* network operators.

In our solution, the UAV and the control station select the network operator based on short-term measurements performed in each one of the operator’s networks. Therefore, we assume two different links and three different routing paths (three network operators). Figure 2 shows the connectivity scenario considered in our approach for *N* Network Operators.

Each agent (UAV and control station) selects independently (i.e., without any information from the other agent) the Operator’s network that assures highest quality of the transmission is the agent’s link: From UAV to antenna or from control station to antenna. A posteriori, another external agent uses this information to select the best routing in the two paths subject to the use of the same operator’s network.

Each agent performs measurements of the network during the first minute of the connection. This time is relatively low in comparison to flight duration. During this time the measurements are stored and the throughput during the next 20 min (assumption of flight average time) is estimated based on those measurements. Such an estimation is performed by using Machine Learning (ML) algorithm, which is trained by real data measurements performed in the network previously.

Once, the system estimates the throughput in both links, then another ML algorithm decides which one of the routing options (which network operator) should be selected in both links (the same network operator). For this, the third ML receives information about the throughput in UAV and control station links (provided by the first two Machine Learning algorithms).

The problem then, consists of solving multi-path routing in two independent links subject to some conditions imposed to the final routing.

Let us remark that the solution provided is valid for any kind of scenario where routing is divided in several links that provide different and independent routing rules, but final routing selection is subject to some conditions that have impact in partial (links) routing. In the case of multiple Radio Access Technology (known as multi-RAT), which is taking increasing importance in 5G networks, the network and the end user are responsible of the selection of the technology and both of them may veto any technology because of, e.g., security reasons. Also in the case of multi-RAT, our solution based on two-step routing decision process is valid.

## 4. Formal Description of the Solution

The formal description of the problem is represented by two agents working on cooperative mode: *a*_1_ (UAV) and *a*_2_ (control station), however, each one of them taking independent decision, so the cooperation is only at the end of the decision making process. The Utility of the final action (decision), *U*(*d*), is a function of the Estimated Utilities of both agents, as shown in (1):

(1) $$U\left(d\right)=f\left(EU_{a_{1}},EU_{a_{2}}\right),$$

where $EU_{a_{1}}$ is the Estimated Utility of state *s* (when the connection will finish) of the agent *a*_1_ in any of the *r* = *r*_1_, … *r_R_* potential routings, i.e.,:

(2) $$EU_{a_{1}}=\{\begin{array}{c} EU_{a_{1}}(s|r_{1})\\ \vdots \\ EU_{a_{1}}(s|r_{R}) \end{array}$$

and $EU_{a_{1}}\left(s|r_{1}\right)$ is the sum of all the potential outcomes at state *s* weighted by the probability that given outcome will appear:

(3) $$EU_{a_{1}}\left(s|r_{1}\right)=\underset{out}{\sum }Outcome\left(s\right)=out\times P(Outcome\left(s\right)=out|r_{1}),$$

*out* being a vector of network parameters defining the connection at state *s*.

At that point, the decision about routing can be taken separately and autonomously provided that we define properly the Utility function *U*(*d*). Therefore, different Machine Learning (ML) algorithms may provide the *EU* at UAV and at the control station.

As we saw in previous sections, the monitoring tools at the UAV (monitoring the radio connections between UAV and antenna) and at the control station (monitoring the radio connection between antenna and control station) provide different parameters and different constrains (for example, round-trip delay must be under given threshold) to select the best routing. Therefore, we can define EU at *a*_1_ and *a*_2_ as a function of the parameters and constrains:

*EU_a_*_1_ = f1(parameter_f1_1, parameter_f1_2, … subject to constrain_f1_1, constrain_f1_2, …)

*EU_a_*_2_ = f2(parameter_f2_1, parameter_f2_2, … subject to constrain_f2_1, constrain_f2_2, …)

And *U*(*d*) is defined as follows:

*U*(*d*) = g[f1(parameter_f1_1, parameter_f1_2, … subject to constrain_f1_1, constrain_f1_2, …); f2(parameter_f2_1, parameter_f2_2, … subject to constrain_f2_1, constrain_f2_2, …)]

The best Utility function, *U*(*d*), is the one that achieves the same final results for decision *d* as in the case when a central system takes the decisions based on complete information [24]. So *max U*(*d*) may be defined as in formula (4).

(4) max U(d)=f(parameter_f1_1,parameter_f1_2,…, parameter_f2_1,parameter_f2_2,…subject to constrain_f1_1,constrain_f1_2,…,constrain_f2_1,constrain_f2_2,…)

The solution of the functions are, in general, *NP*-complete, so finding the function *g* that maximizes *U*(*d*) is not solvable with reasonably limited time. However, if we have a number of potential functions *G* = {*g*_1_, *g*_2_, …, *g_K_*}, then we can empirically select the best function, understood as the function that provides best results of the final decision. This means that the best function *g_i_* ∈ *G* will provide those results, which are closest to max *U*(*d*) as defined in (4). Let *G* be the hypothesis space, with *g*_1_, …, *g_K_* being each of the hypothesis of the hypothesis space. Then the final *U*(*d*) can be calculated as follows:

(5) $$U=\underset{g_{i}\in G}{\arg\;\min}||g_{i}\left[f_{1},f_{2}\right],\max U\left(d\right)||$$

where ||.|| is the distance between the results (whichever the output parameters are) of the two functions.

**Figure 3:**
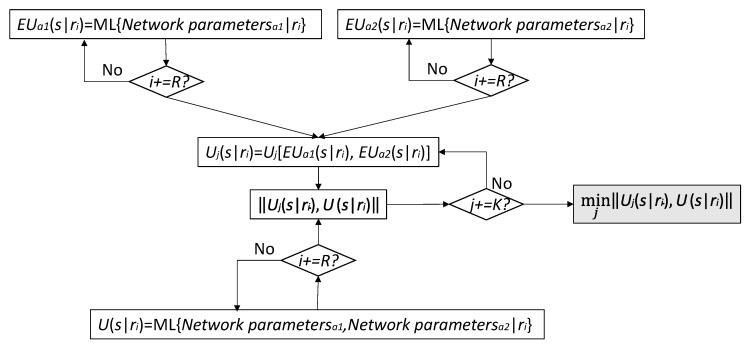
Machine Learning (ML)-based methodology for achieving final Utility functions used in two-steps routing selection.

As far as the Utility will be estimated by using ML, then the steps to achieve the final functions are shown in Figure 3.

## 5. Test Results

The tests provided in this section are related with the two-step ML-based solution presented in previous section. Concretely, we performed multiple measurements in LTE network and, based on those measurements, we trained the two ML algorithms (ML_1_ and ML_2_), each algorithm representing one link of the network (i.e., UAV-antenna and control station-antenna). ML_1_ and ML_2_ use the same models for resource usage prediction, however, since both links are measured using different parameters, then ML_1_ and ML_2_ will tune the layers in a different way. In addition, another algorithm, ML_3_, is trained using measurements of both links, this is end-to-end measurements from UAV to control station. Moreover, we assume that the selection of the routing in link 1 and link 2 are subject to the following constrain: The connection (routing path) in link 1 and in link 2 must be served by the same operator in order to avoid inter-operator roaming (IPX network, see Figure 2). ML_3_ has the end-to-end information from link 1 and 2, so the final routing selected by ML_3_ is the optimal one. ML_3_ corresponds to the best trained machine (named *f* in the previous section).

The next step consists of setting a hypothesis space *G* with two potential functions and check which one of them fits better with function *f*. The best function in *G* is considered the Utility function and will be used during the evaluation phase.

The evaluation phase follows the footsteps of Machine Learning theory, so the measurements from the network are classified in different sub-groups and k-fold cross-validation is performed [25,26,27].

### 5.1. Measurements of the Network Links

As presented above, the measurements of the network at the beginning of the connection (1 min-duration) should provide information about the functioning of the network during the whole connection between UAV and control station (20-min duration). In short, the input data of the machine learning algorithms are information of the links during 1 min. This information is used by the resource-usage prediction models of the Machine Learning for predicting the throughput available for the connection during the next 20 min (estimated flight duration). In order to train the ML, the total throughput of the links is also measured in the links 1 (UAV to antenna) and 2 (control station and antenna).

The link 1 (between the UAV and the antenna) has been measured by monitoring at the UAV the data throughput (Data Throughput Info Message CMD TYPE: 0 × 60) and the round-trip delay of signaling messages. For this scope, we use the abovementioned X-CAL application with the option of sending LTE_DATA_THROUGHPUT_INFO (0 × 60) messages and, on the other hand, the sending of ICMP messages (ping) for measuring round-trip delays on the link.

After that, the output data are measured by monitoring the network and measuring the mean throughput during 20 min. This is available thanks to the data registered in the chipset (reading operation provided by X-CAL) even if the whole available throughput has not been used by the connection during all the time.

**Figure 4:**
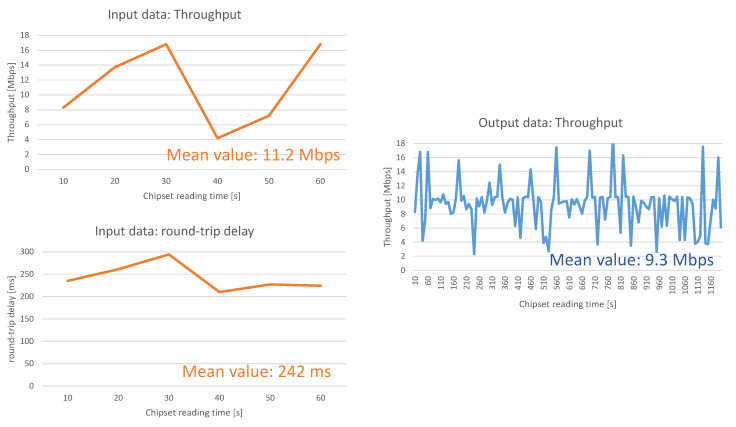
Input and Output data of ML_1_. Input data are throughput and round-trip delay (RTD) (1 min). Output is throughput (20 min). The values of <input1,input2;output> for this concrete sample are: <11.2 Mbps,242 ms; 9.3 Mbps>.

Figure 4 presents one sample of the input and output reading data in link 1 for one operator’s network. The chipset is read each 10 s. The input data are read during the first minute and the output data during 20 min.

The parameters that measure the quality of the connection between control station and antenna (link 2) are the round-trip delay (defined as before), the throughput and TCP packet losses (measured during 1 min). Also in this case, the throughput of the network is measured during the next 20 min in order train properly ML_2_.

In both links, three different LTE operators are measured, so that ML algorithms will estimate the throughput available in each one of the operator’s networks.

### 5.2. ML Algorithms and Hypothesis Space of U(d)

The Machine Learning algorithms ML_1_ and ML_2_, implement in the first layer two well-known resource usage prediction models: An epidemic model defined in [28,29] and a model based on Autoregressive Integrated Moving Average (ARIMA) that assumes that the throughput follows a Gaussian distribution. The ARIMA model that we implemented is presented in Section 3.3 of the paper [30].

**Figure 5:**
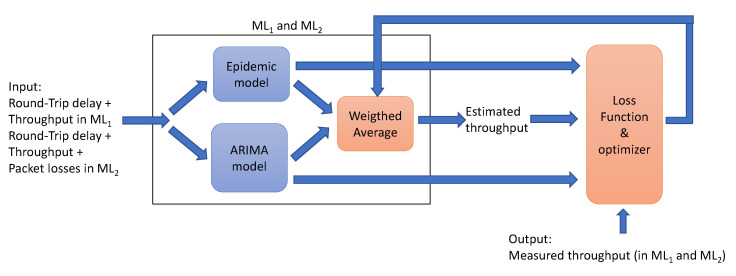
Scheme for ML_1_ and ML_2_.

In the second layer of the ML algorithm, it is implemented a weighted average algorithm where the average value is a parameter that must be trained, see Figure 5. At last the Loss function in training process is the difference of the throughput estimated by the ML and the throughput measured in the next 20 min. If the difference is bigger than δ, then the values of throughput estimated by each one of the models is compared with the measured throughput and the weight of the weighted average formula is corrected to give more weight to the most exact model.

The loss function and optimizer is presented in Algorithm 1.

**Algorithm 1:** Loss Function and Optimizer1: **procedure** LossFunc_Optimizer 2: Δ ← |Estimated_throughput—Output_throughput| 3: **case** δ > Δ   **exit Null** 4: Δ1 ← Epidemic_Model_throughput—Output_throughput 5: Δ2 ← ARIMA_Model_throughput—Output_throughput 6: weight ← Δ1/Δ2 7: **exit** weight

The algorithm minimizes the error of the estimated output by selecting the incidence of epidemic and ARIMA prediction algorithms in the final result.

ML_1_ may adjust different weight for each operator, since each operator may have a different policy or policies that make resource usage prediction behaves in a different way. So, ML_1_ (as well as ML_2_) are trained with only the input data of one operator.

ML_3_ uses the same prediction models, however the input and output data in this case are the measurements including both the links, i.e., from UAV to control station. For this, we send the messages from control station to the UAV instead to the server in the network operator. Since, in the case of ML_3_ training, the measurements are taken in UAV, they are the same as in ML_1_, i.e., data throughput and the round-trip delay of signaling messages, subject to operator in link 1 is the same than operator in link 2 (in order to avoid inter-operator roaming).

Let us remark that the ML_3_ has been created only for selecting the optimal function *U*(*d*), so ML_3_ will be run only during the training phase. Afterwards, during the normal flight of the drone, only ML_1_, ML_2_ and *U*(*d*) will obtain the best routing for each new connection.

At last, the function *U*(*d*) should be selected such that *U*[*f*_1_(ML_1_,),*f*_2_(ML_2_)] ≈ *f*(ML_3_). *U*(*d*) is a function that selects the routing in link 1 and link 2, so, in our case it is the selection of the operator that will serve the connection. The inputs of *U*(*d*) are the estimated values of throughput in link 1 (calculated by ML_1_) and in link 2 (calculated by ML_2_) for each one of the operators: *Est_Thr_link, operator_*. The output of *U*(*d*) is a value from 1 to 3 (one of the three operators). The hypothesis space *G* of potential function *U*(*d*) includes the following hypothesis:

(6) $$G_{1}=\underset{j\in \mathbb{N}}{\arg\;\max}\left[\underset{i\in \mathbb{N}}{\min}(Est\_Thr_{i,j}\right)],\;i=\left[1,\;2\right],j=\left[1,\;3\right]$$

(7) $$G_{2}=\underset{j\in \mathbb{N}}{\arg\;\max}\left[\frac{1}{2}\times \sum_{i\in \mathbb{N}}\left(Est\_Thr_{i,j}\right)\right],\;i=\left[1,\;2\right],j=\left[1,\;3\right].$$

*G*_1_ and *G*_2_ are the boundaries of the weighted function (8):

(8) $$\underset{j\in \mathbb{N}}{\arg\;\max}\left[w_{1}\times \underset{i\in \mathbb{N}}{\min}\left(Est\_Thr_{i,j}\right)+\left(1-w_{1}\right)\times \underset{i\in \mathbb{N}}{\max}\left(Est\_Thr_{i,j}\right)\right].$$

In a general case, the hypothesis space *G* should consider all the policies for routing that consider relations of the two links.

### 5.3. Test Methodology

To sum up previous sections, the experiments have been provided with the next assumptions:

(1) Two Machine Learning algorithms for both agent 1 (e.g., UAV) and agent 2 (e.g., control station), called ML_1_ and ML_2_, respectively. ML_1_ searches the best routing (network operator that will serve the connection) in link 1 between UAV and antenna, whereas ML_2_ finds the best routing (i.e., which network operator will serve the connection) in link 2 between control station and antenna;
(2) 3 potential routings (3 operators) in agent 1 (e.g., UAV to antenna) and in agent 2 (e.g., control station to antenna);
(3) routing in agent 1 is selected based on round-trip delay (RTD) and data throughput measurements in link 1 (between UAV and antenna). Routing in agent 2 is selected based on RTD measurements, data throughput and packet losses performed in link 2 (between control station and antenna);
(4) a third Machine Learning algorithm (ML_3_) calculates the best routing in link 1 and 2, so the best end-to-end routing. The inputs of ML_3_ are round-trip delay and data throughput measured in end-to-end connections (between UAV and control station). This corresponds to the function max *U*(*d*) defined in Section 4; and
(5) a Utility function is considered such that quasi-optimal end-to-end routing can be selected based on partial link 1 and link 2 routings. Then, with the results of ML_1_ and ML_2_ and assuming an appropriate Utility function, the system is able to select quasi-optimal end-to-end routing. The optimal Utility function will be the one that better approximates quasi-optimal solution (from partial link 1 and link 2 routings together with Utility function) and end-to-end routing.

The steps realized in the tests are the following: (1) Validation of ML_1_, (2) validation of ML_2_, (3) validation of ML_3_, and (4) selection and validation of *U*(*d*).

Since the number of measurements in the network is limited (150 measurements in link 1, link 2 and end-to-end), we introduced *k*-fold cross-validation for validating the Machine Learning algorithms. The *k*-fold cross-validation assumes that data are divided in *k* groups (in our case *k* = 3 with 50 samples per group). The validation is then performed *k* times, leaving *k*−1 groups of data for training and one group for validating. From one test to the next one, the group of data selected for validation will change. The final results of the validation is the average of the *k* tests.

At last, the selection of *U*(*d*) consists of comparing the end-to-end estimated throughput with the estimated throughputs in link 1 and 2. Therefore, we need a last dataset composed of measurement samples taken at the same time in link 1, 2, and end-to-end. This dataset is composed by 300 samples (100 for each operator) with the next measurements: Round-trip delay in link 1, throughput in link 1, round-trip delay in link 2, throughput in link 2, packet loss ratio in link 2, round-trip delay in end-to-end connection, and throughput in end-to-end connection.

### 5.4. Test Results

When analyzing the prediction models used in ML_1_, ML_2_, and ML_3_, we may observe that the epidemic model overestimates the throughput whereas the Autoregressive Integrated Moving Average model underestimates the throughput. It is a suitable situation for introducing a weighted average mechanism, which corrects over- and underestimation.

**Figure 6:**
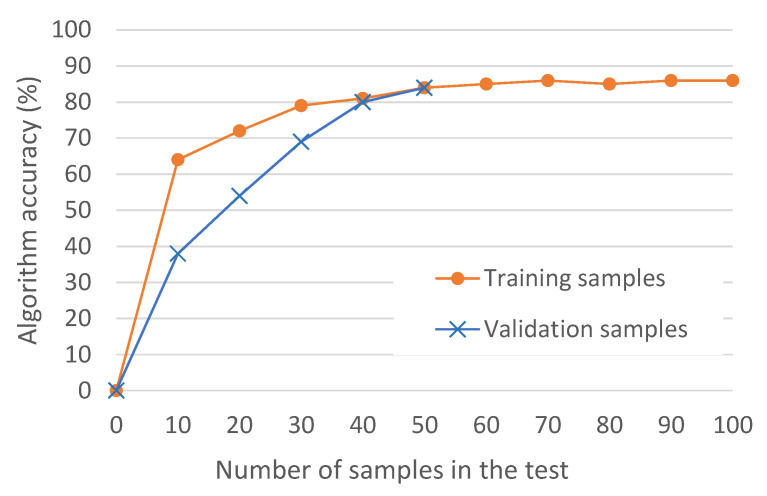
Accuracy of ML_1_ for increasing number of training samples and validation samples.

The first results (see Figure 6) show that the ML overfits results for up to 30 training samples. If the samples go beyond 30, then the ML accuracy is similar in training and in validating phases. One sample is considered positive in the case that the estimated (by the algorithm) throughput and the measured throughput differ by less than 5%, i.e.,: Est_thr ϵ [0.95xMeas_thr, 1.05xMeas_thr]. Moreover, it is important to remark that Figure 6 is based on one test containing 150 training samples and 50 validation samples (in this case we use holdout cross-validation instead of *k*-fold cross-validation)

The second important conclusion of Figure 6 is that the results achieve around 86–87% accuracy. The reason that the algorithms does not reach higher accuracy is due to the variability of throughput in the commercial network, as shown previously in Figure 4.

Table 1 shows the differences of accuracy (of validation samples) between ML_1_, ML_2_ and ML_3_. These results are based on 3-fold cross-validation methodology and the confidence intervals presented in the results are based only on three measurements.

The input data of ML_1_ and ML_2_ are different, so one could expect that the results differ between the two algorithms, however the table shows that the results are very similar. We may conclude that the gain of having a higher number of parameters in the prediction models is negligible in comparison to the gain of having two prediction models properly “weighted”. On the other hand, ML_3_ achieves also similar accuracy than ML_1_ and ML_2_. ML_3_ estimates the throughput in end-to-end path containing radio links (UAV-antenna and antenna-control station) and fixed links (antenna-core-antenna, i.e., fronthaul and backhaul). The fact that the results are similar in end-to-end path means that the variability of the network (which decreases the accuracy) is present, most of all, in the radio links.

The last tests aim to define which utility function of the hypothesis space defined above is the optimal for estimating the end-to-end throughput. The first step is to train ML_1_, ML_2_ with all the 150 measurement samples obtained in link 1 and link 2. In this test we assume that ML_1_, ML_2_, and ML_3_ do not need to be validated, so all the data can be used for training the machines. Afterwards, ML_3_ is trained with the 150 samples of end-to-end connection.

With the 300 measurements (100 samples/operator) taken at the same time in link 1, link 2 and end-to-end connection, we estimate the throughput in link 1 (by using the trained ML_1_), in link 2 (by using the trained ML_2_) and in end-to-end connection (by using the trained ML_3_). Table 2 presents the estimated values of throughput for one example sample. Let us remark that the throughput is measured in uplink direction.

For this concrete example sample, end-to-end routing would be selected by using Operator 2 (11.1 Mbps is the biggest throughput in end-to-end connection). On the other hand, *G*_1_ is equal to 11.3 Mbps, see formula (4), and the Operator selected by *G*_1_ is Operator 2; whereas *G*_2_ is equal to 14.1 Mbps, see formula (5), and the Operator selected by *G*_2_ is also Operator 2. In this example the values of throughput for Operator 2 are really much better than for other Operators, so it is logic that all the functions aiming to maximize the throughput will select Operator 2.

By estimating the throughput of the 300 validation samples (100 per Operator), we get similar conclusions, the throughput of *G*_1_ is closer to the throughput estimated by ML_3_ in the end-to-end connection in almost all the cases and the two Utility functions, *G*_1_ and *G*_2_, select the same Operator chosen by ML_3_. The reason of this almost unanimity of results is that in all cases link 1 is much worse than link 2, and, because of this, the values of end-to-end connection throughput are similar to link 1 throughputs (bottleneck). In this situation, *G*_1_ is securely the best hypothesis of the hypothesis space *G*.

So, in normal functioning of the routing selection tool, ML_1_ and ML_2_ will estimate (after training) the value of throughput for each Operator and *G*_1_ will determine the estimated end-to-end throughput. Based on this estimations, *G*_1_ will select the best end-to-end Operator.

## 6. Conclusions

This paper discusses the reliability of the communication between the unmanned aerial vehicle and the control station when such a communication uses the infrastructure of commercial mobile networks. The main contribution of the paper is a methodology to provide two-phase selection algorithm based on learning machines. The two phases consist of two independent selection processes that are a posteriori connected with a Utility function that optimizes the final selection. The idea is to get final results that are as close as possible to the result that would be selected by a hypothetical algorithm that would own all the information that are available at each phase.

With the presented algorithm, the UAVs will select the mobile network operator that will serve the connection during the flight and, on the other hand, the control station will select also the operator for the connection based on the parameters of the radio links between the control station and the antenna. A later selection will take into consideration the two links and will take the final decision on the network operator that will connect the UAV with the control station.

The results show that it is possible to optimize two-phase selection process based on Machine Learning.

## Figures and Tables

**Table 1:** Accuracy of the ML algorithms for 3 tests following 3-fold cross-validation.

Machine Learning Algorithm	Accuracy
ML_1_ (UAV to antenna)	84% ± 2%
ML_2_ (control station to antenna)	82% ± 3%
ML_3_ (UAV to control station)	81% ± 3%

**Table 2:** Estimated values of throughput in link 1, link 2, and end-to-end connection.

Link	Estimated Throughput [Mbps]		
	Operator 1	Operator 2	Operator 3
Link 1—ML_1_	8.3	11.3	10.6
Link 2—ML_2_	14.7	16.8	15.4
End-to-end—ML_3_	9.1	11.1	10.8

## Data Availability

Data sharing not applicable.
